# An Alternatively Spliced Variant of METTL3 Mediates Tumor Suppression in Hepatocellular Carcinoma

**DOI:** 10.3390/genes13040669

**Published:** 2022-04-11

**Authors:** Rui-Yao Xu, Zhan Ding, Qing Zhao, Tiao-Ying Ke, Shu Chen, Xing-Yu Wang, Yao-Yun Wang, Meng-Fei Sheng, Wei Wang, Ni Long, Yu-Xian Shen, Yong-Zhen Xu, Wei Shao

**Affiliations:** 1 School of Basic Medical Sciences, Anhui Medical University, Hefei 230032, China; xuruiyao@stu.ahmu.edu.cn (R.-Y.X.); zhaoqing@stu.ahmu.edu.cn (Q.Z.); ketiaoying@stu.ahmu.edu.cn (T.-Y.K.); shuchen976@gmail.com (S.C.); wangxingyu@stu.ahmu.edu.cn (X.-Y.W.); 2145010060@stu.ahmu.edu.cn (Y.-Y.W.); 2145010057@stu.ahmu.edu.cn (M.-F.S.); shenyx@ahmu.edu.cn (Y.-X.S.); 2 Anhui Provincial Laboratory of Microbiology and Parasitology, Department of Microbiology and Parasitology, Anhui Medical University, Hefei 230032, China; 3 State Key Laboratory of Virology, Hubei Key Laboratory of Cell Homeostasis, College of Life Science, Wuhan University, Wuhan 430072, China; dingzhan@cemps.ac.cn (Z.D.); 2019202040138@whu.edu.cn (N.L.); 4 Department of General Surgery, The First Affiliated Hospital of Anhui Medical University, Hefei 230032, China; wwok68@hotmail.com; 5 Biopharmaceutical Research Institute, Anhui Medical University, Hefei 230032, China

**Keywords:** METTL3, splice variants, RNA m^6^A, hepatocellular carcinoma, tumor suppressor

## Abstract

Many post-transcriptional mRNA processing steps play crucial roles in tumorigenesis and the progression of cancers, such as N6-methyladenosine (m^6^A) modification and alternative splicing. Upregulation of methyltransferase-like 3 (METTL3), the catalytic core of the m^6^A methyltransferase complex, increases m^6^A levels and results in significant effects on the progression of hepatocellular carcinoma (HCC). However, alternative splicing of METTL3 has not been fully investigated, and the functions of its splice variants remain unclear. Here, we analyzed both our and online transcriptomic data, obtaining 13 splice variants of METTL3 in addition to canonical full-length METTL3-A in HCC cell lines and tissues. Validated by RT–qPCR and Western blotting, we found that METTL3-D, one of the splice variants expressing a truncated METTL3 protein, exhibits higher levels than METTL3-A in normal human livers but lower levels than METTL3-A in HCC tumor tissues and cell lines. Further functional assays demonstrated that METTL3-D expression decreased cellular m^6^A modification, inhibited the proliferation, migration, and invasion of HCC cells, and was negatively associated with the malignancy of patient tumors, exhibiting functions opposite to those of full-length METTL3-A. This study demonstrates that the METTL3-D splice variant is a tumor suppressor that could potentially be used as a target for HCC therapy.

## 1. Introduction

RNA plays a central role in gene expression, serving as a vital link for genetic information passing from DNA to protein [1]. The maturation of messenger RNA precursors requires removing introns and ligating exons, processes catalyzed by the spliceosome and named pre-mRNA splicing [2,3]. In humans, more than 95% of nascent transcripts have been alternatively spliced, generating multiple mRNA isoforms (splice variants) from one gene [4]. Splice variants are different in their coding sequences and/or untranslated regions (UTRs), in which approximately 60–75% are within the coding regions that provide protein diversities [2,5]. Alternative splicing (AS)-affected tumor biology includes metabolism, apoptosis, cell cycle control, invasion, and metastasis [6,7]. Splice variants of many oncogenes are associated with the occurrence and development of cancers, such as breast cancer, prostate cancer, cervical cancer, and hepatocellular carcinoma (HCC) [8,9,10,11]. AS changes are prevalent in HCC, and many are associated with HCC prognosis [12,13]; therefore, splice variants are sources of novel biomarkers and therapeutic targets for HCC [12,14]. For example, RCAN1.4, a splice variant of the regulator of calcineurin 1 (RCAN1), inhibits the proliferation, migration, and invasive activity of HCC cells [15]. CD73S, a shorter splice variant of CD73, exhibits functional differences from the canonical isoform CD73L in modulating the proliferation potential of HCC cells [16]. PRRL, a splice variant of Numb encoding a longer proline-rich region, is upregulated in HCC and associated with early recurrence and reduced overall survival after surgery, exhibiting an opposite function to the Numb-PRRS isoform in the proliferation and invasion of HCC cells [17,18].

Post-transcriptional regulation of mRNAs also includes numerous modifications of ribonucleotides with crucial roles in the functional regulation of genes. m^6^A is the most abundant modification in eukaryotic mRNAs [19,20]. In 2012, a study combining m^6^A-specific methylated RNA immunoprecipitation with next-generation sequencing (MeRIP-Seq) profiled the transcriptome-wide m^6^A distribution in humans and mice, revealing that m^6^A sites are particularly enriched near stop codons and in the 3′-UTRs with a consensus sequence of RRACH [19,21]. During the past few years, m^6^A modification in mRNAs has played a critical role in mammals, such as embryonic development, neurogenesis, circadian rhythm, sex determination, and human carcinogenesis [22,23,24,25].

m^6^A modification is catalyzed by a multicomponent enzyme complex collectively termed m^6^A “writers”, including methyltransferase-like 3 (METTL3), methyltransferase-like 14 (METTL14), Wilms tumor suppressor 1-associated protein (WTAP), zinc finger CCCH domain-containing protein 13 (ZC3H13), and methyltransferase-like 16 (METTL16) [20]. The occurrence of m^6^A modification requires at least two separate proteins, MT-A and MT-B [26]. The 200-kDa MT-A is a multimeric protein containing a 70-kDa AdoMet-binding subunit MT-A70, which has been identified in eukaryotes and prokaryotes [27]. MT-A70 is a crucial subunit of (N6-adenosine)-methyltransferase, and both METTL3 and METTL14 are members of the MT-A70 family [28]. METTL3 plays a pivotal role in the posttranscriptional methylation of internal adenosine residues in eukaryotic mRNAs. Silencing METTL3 results in significantly reduced peaks of m^6^A in mouse embryonic stem cells, HeLa cells, and HepG2 cells [29,30]. METTL14, together with METTL3, forms a stable heterodimer of the methyltransferase complex that mediates cellular m^6^A deposition on mammalian mRNAs. Disruptions of METTL3 and METTL14 affects plant growth, yeast meiosis, body mass and metabolism, synaptic signaling, circadian clock regulation, and stem cell self-renewal and differentiation [29]. Many studies have shown that METTL3 promotes tumor growth and aggressiveness by regulating the mRNA decay and stability or mRNA translation of numerous tumor-associated genes, such as SOCS2, BATF2, EGFR, TAZ, MAPKAPK2, and DNMT3A [31,32,33]. METTL3 was observed to colocalize with splicing factors in nuclear speckles, indicating a potential regulatory role of m^6^A in mRNA metabolism [34]. Additionally, alternative splicing changes have been found in METTL3- and METTL14-knockdown cells, suggesting that m^6^A modification is involved in pre-mRNA splicing [35].

Liver cancer is a serious global cancer and has a high risk of cancer-related death [36]. HCC is one of the most frequently occurring primary malignant liver tumors and understanding the molecular mechanism of HCC development is crucial. Many studies have demonstrated the potential implications of m^6^A modification in diagnosing and treating liver cancer [37]. METTL3 was found to be upregulated in hepatocellular carcinoma (HCC), and METTL3 knockout notably suppressed hepatocellular carcinoma tumorigenicity in vivo [31]. METTL3 overexpression is associated with a poor prognosis in patients with hepatocellular carcinoma [38]. Additionally, patients with high METTL3 expression show a poor overall survival and poor disease-free survival [31].

Although METTL3 has been studied extensively in recent years, the existence and function of its splice variants have not been reported. In this study, METTL3 splice variants were identified and analyzed in normal liver and HCC tissues. The METTL3-B variant is conserved in humans and mice, and the METTL3-D variant is a functionally distinct isoform compared with the canonical full-length METTL3-A. The mRNA and protein levels of METTL3-D were significantly decreased in HCC tumor tissues. The proliferation, migration and invasion of HCC cells were inhibited when METTL3-D was overexpressed. METTL3-A and METTL3-D oppositely controlled the expression of METTL3 downstream target genes. Finally, high levels of METTL3-D were significantly associated with early HCC tumor stages and longer overall survival times, suggesting that METTL3-D is a tumor suppressor in HCC.

## 2. Materials and Methods

### 2.1. Cell Culture

The human liver cancer cell lines HepG2, MHCC97-L, and HCCLM3 were acquired from American Type Culture Collection, Manassas, VA, USA. All cell lines were cultured in Dulbecco’s modified Eagle’s medium (DMEM; Invitrogen, CA, USA) supplemented with 10% fetal bovine serum (FBS; Invitrogen, CA, USA) and antibiotics (100 IU/mL penicillin and 0.1 mg/mL streptomycin, Sigma-Aldrich, St Louis, MO, USA) at 37 °C in an incubator with 5% CO_2_.

### 2.2. Human Specimens

HCC patient samples from the First Affiliated Hospital of Anhui Medical University (Hefei, China) were stored in a −80 °C freezer after collection. This study was approved by the Ethics Committee of Anhui Medical University. The clinical specimens were used in accordance with the Declaration of Helsinki. The clinical characteristics of all the patients are listed in Table S1.

### 2.3. Plasmid Transfection and Antibodies

Plasmids for the expression of Flag-tagged METTL3-A (WT) and variants (-B, -C, and -D) were constructed using the pcDNA3 vector. HCC cell lines were transiently transfected with the plasmids and jetPRIME transfection reagent (PolyPlus-transfection^®^, Illkirch, France), and the total RNA and proteins were extracted 48 h later. The total proteins were extracted from cells or tissues with RIPA lysis buffer (Beyotime, Shanghai, China). Equal amounts of proteins were separated with 10% SDS-PAGE gel and transferred to polyvinylidene difluoride (PVDF) membranes (Millipore, Bedford, MA, USA). The membranes were blocked with 5% nonfat milk at room temperature for 1.5 h and then incubated overnight at 4 °C with primary antibodies: anti-GAPDH (1:4000, E-AB-20059, Elabscience, Houston, TX, USA), anti-FLAG antibody (1:4000, F2555, Sigma-Aldrich, St Louis, MO, USA), or anti-METTL3 antibody (1:1000, ab#195352, Abcam, Cambridge, UK). The primary antibodies were detected with horseradish peroxidase (HRP) conjugated goat anti-rabbit IgG (1:8000, #AS014, Abclonal, Wuhan, China) or goat anti-mouse IgG (1:8000, #AS003, Abclonal, Wuhan, China), and the secondary antibodies were visualized by ECL Prime Western blotting Detection Reagents. Primary (P0023A; Beyotime Biotechnology, Shanghai, China) and secondary (P0023D; Beyotime Biotechnology, Shanghai, China) antibody dilution buffers were used to dilute the corresponding antibodies.

### 2.4. PCR and Sequencing

cDNAs of METTL3 splice variants were amplified using specifically designed primers listed in Table S2. Sanger sequencing was performed by Qing-ke Company, and the sequences were aligned by Chromas (Technelysium Pty Ltd., Tewantin, Australia). Next-generation RNA sequencing was performed on an Illumina platform with 150 bp paired-end reads. Raw reads were filtered by FastQC v0.11.8 and TrimGalore v0.6.2, and the clean reads were then mapped to the human genome (GRCh38) using STAR v2.6.1a [39].

### 2.5. Quantitative Real-Time PCR

Total RNA was extracted from HCC patients and HCC cells using TRIzol reagent (Invitrogen, CA, USA) and then stored at −80 °C until use. Reverse transcription was performed using SuperScript III Reverse Transcriptase (Life Technologies, Carlsbad, CA, USA). Quantitative PCR was performed using an Applied Biosystems StepOnePlus Real-Time PCR System with SYBR Premix Ex TaqTM II (TaKaRa, Otsu, Shiga, Japan), and the data were analyzed using the 2-ΔΔCq method [40] and normalized to glyceraldehyde 3-phosphate dehydrogenase (GAPDH).

### 2.6. Bioinformatics and Online Resource

The Ensembl gene browser database [41] was used to query specific METTL3 variants. Sequences of cDNAs and amino acids were also downloaded from Ensembl. The levels of METTL3 splice variants in human tissues were obtained from the Genotype-Tissue Expression (GTEx) project and analyzed using GTExPortal (https://www.gtexportal.org/home/) (accessed on 5 November 2020).The sequencing data of HepG2 cells from the PacBio platform in ENCODE (ENCSR834DQL, https://www.encodeproject.org/files/ENCFF470UHX/) (accessed on 13 July 2020) were downloaded, and the transcripts were visualized using JBrowse [42,43]. Isoform levels and patient survival analyses were performed using GEPIA2 [44].

### 2.7. RNA m^6^A Quantification

The levels of RNA m^6^A in HepG2 cells were detected by dot blots using an anti-m^6^A antibody (#517924; Zhengneng Biotechnology, Chengdu, China), and the signal densities were analyzed by ImageJ software (National Institutes of Health, Bethesda, MD, USA). The levels of RNA m^6^A from patients were measured using an EpiQuik m^6^A RNA methylation quantification kit (P9005; Epigentek, Farmingdale, NY, USA).

### 2.8. Cell Growth and Proliferation Assays

Twenty-four hours after transfecting METTL3 expression plasmids, HCCLM3 or HepG2 cells were seeded at 5 × 10^3^ cells per well in 24-well plates and cultured for 5 days. Cell numbers were counted every day using a hemocytometer.

CCK-8 assays (Beyotime, Shanghai, China) were performed to determine cell proliferation. Briefly, 100 μL of 2 × 10^3^ cells was seeded into each well of the 96-well plate and incubated in a 5% CO_2_ incubator at 37 °C for 0, 24, 48, and 72 h. Next, 10 μL of CCK-8 solution was added to each well and incubated for 2 h. The absorbance was measured at 450 nm using a spectrophotometer.

MTT assays were performed using an MTT Cell Proliferation Assay Kit (Beyotime, Shanghai, China). Briefly, 100 μL of 5 × 10^3^ cells was seeded into each well of the 96-well plate and incubated for 0, 24, 48, and 72 h at 37 °C, and then 10 μL of MTT stock solution was added for another 4 h of incubation. The 96-well plate was then foil wrapped after adding 100 μL of DMSO solution, followed by shaking on an orbital shaker for 15 min to fully dissolve the MTT formazan. Absorbance was measured at 490 nm using a spectrophotometer.

### 2.9. Cell Migration and Invasion Assays

The migration of HCC cells was detected using the wound-healing assay. For the cultured six-well plate, a linear wound was created using a 200-μL pipette tip, and two photographs were taken at the 24 h and 48 h time points. The wound area was measured using ImageJ software [45].

The invasion ability of cells was determined using a Transwell chamber inserted with 8-µm pore size polycarbonate membranes (Corning, NY, USA). Briefly, 5 × 10^4^ cells in serum-free DMEM were plated into the Transwell upper chamber and coated with Matrigel (BD Biosciences, San Jose, CA, USA), DMEM with 20% FBS was added to the lower chamber. After 24 h of incubation at 37 °C with 5% CO_2_, the non-invading cells sticking to the top chamber were cleared using a cotton swab, and the invading cells that had migrated to the lower surface of the membrane were fixed with 20% precooled methanol for 30 min and stained with 0.5% crystal violet (Servicebio, Wuhan, China) for 10 min. The Transwell membranes containing stained cells were digitally imaged and then counted using ImageJ software [45].

### 2.10. Statistical Analysis

All the data were analyzed by GraphPad Prism as means ± SD to assess group differences. Statistical significance was defined by Student’s *t* test, and a *p* value < 0.05 was considered to be statistically significant.

## 3. Results

### 3.1. Differentially Spliced Variants of METTL3 in Human Tissues and HCC Cell Lines

**Figure 1:**
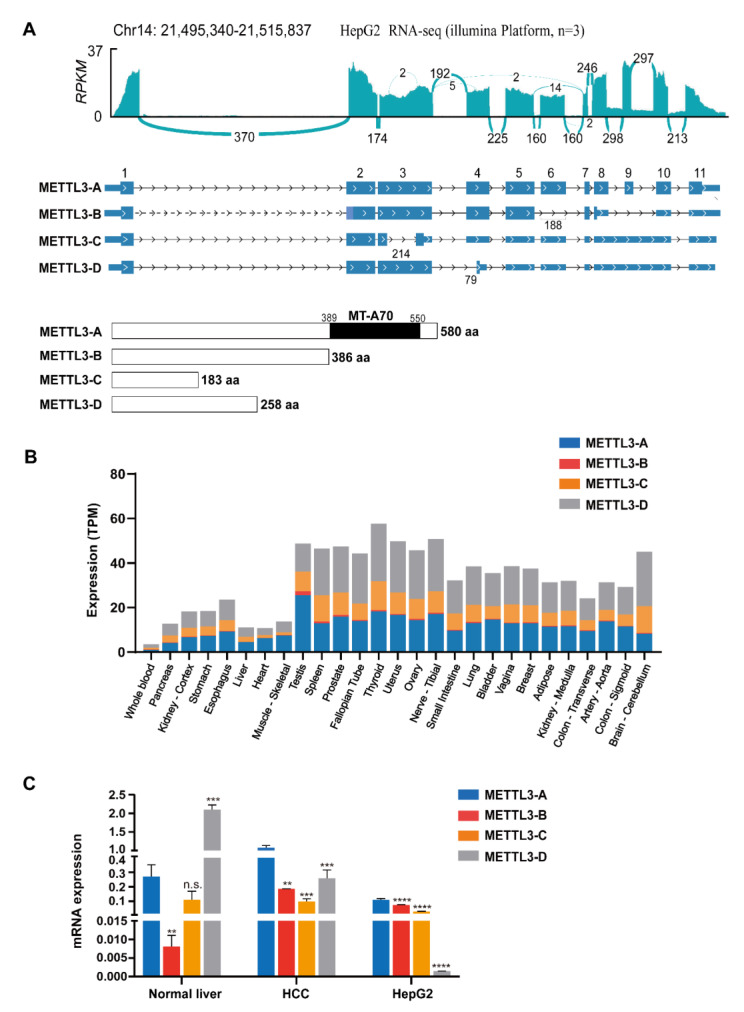
Splice variants of METTL3 in human tissues and HCC cell lines. (**A**) Analyses of alternative splicing of METTL3 using online and our transcriptomic data. Upper, Sashimi plot of METTL3 using the aligned RNA-seq reads from HepG2 cells. Middle, comparison of the four METTL3 splice variant transcripts. The data were downloaded from Ensembl. Lower, comparison of the METTL3 splice variant protein domains. (**B**) mRNA levels of METTL3-A, B, C, and D in human tissues. Analyses were performed using data from GTExPortal. (**C**) mRNA levels of METTL3-A, -B, -C, and -D in human normal liver, HCC tumor tissues, and HepG2 cells. The data are expressed as means ± SD. ** *p* < 0.01, *** *p* < 0.001, **** *p* < 0.0001 (*t* test).

The human METTL3 gene is located on Chr14; it has 11 canonical exons and is alternatively spliced, similar to most other mammalian genes [4]. To fully investigate the splicing variants of METTL3 in HCC, we first analyzed our next-generation RNA-seq data of the HCC cell line HepG2. Including METTL3-A, the full-length and canonically spliced product, fourteen transcripts from the METTL3 gene in HepG2 cells were constructed, all of which can be found in the online *Ensembl* database (Figure 1A upper and Figure S1A).

Considering their abundance, length of the transcript and coding region, and conservation in mammals, we chose three splice variants (METTL3-B, -C, and -D) and full-length METTL3-A for further investigation. In the variant METTL3-B, exons 6 and 9 were skipped, and the skipping of exon 6 introduced a premature termination codon (PTC) resulting in the translation of a 386-aa peptide without the MT-A70 domain. In variant C, the middle part of exon 3 was missed, and introns 8 and 9 were totally retained, introducing an even earlier PTC that allows METTL3-C to be translated into a much smaller protein. Two alternative splicing events were found to generate the variant METTL3-D, alternative 3′SS to generate a shorter exon 4 (here named exon 4a) and the retention of introns 8 and 9; thus, METTL3-D could be translated into a 258-aa peptide without the C-terminal catalytic domain (Figure 1A lower and Figure S2). Next, we analyzed the RNA levels of METTL3 splicing variants in human tissues using GTExPortal. All fourteen isoforms were detected in human tissues with certain specificities (Figure S1B). Further comparison showed that METTL3-A, -C, and -D were relatively higher in all the analyzed tissues, while METTL3-B was relatively low. Surprisingly, METTL3-D was at similar or even higher levels than METTL3-A in most human tissues (Figure 1B).

To confirm the presence of these splice variants, we verified the existence of METTL3-A, -C, and -D by RT-PCR using specific primers and Sanger sequencing (Figure S3A) and METTL3-A and -D by detecting full transcripts using public HepG2 third-generation sequencing data (Figure S3B). Among those variants, METTL3-B is the only conserved variant between humans and mice (Figure S4A,B. Failing to verify the specific exonic junctions of METTL3-B in human cells could be due to its low level; however, we successfully detected Mettl3-b in the mouse sample (Figure S4C).

Because canonical full-length METTL3-A regulates the carcinogenesis, tumor progression, and drug resistance of HCC [31,46], we carefully detected the levels of METTL3 splicing variants in normal human liver, HCC tumor tissues, and HepG2 cells using RT-qPCR. Consistent with the above GTExPortal data, the level of METTL3-D in normal human liver was much higher than that of METTL3-A (Figure 1C left). By contrast, METTL3-D was significantly decreased in the tumor tissues and HepG2 cells (Figure 1C middle and right). Additionally, METTL3-B expression was increased in HCC tissues and cells, while METTL3-C expression was at similar levels.

### 3.2. METTL3-D Is Negatively Associated with HCC Malignancy

**Figure 2:**
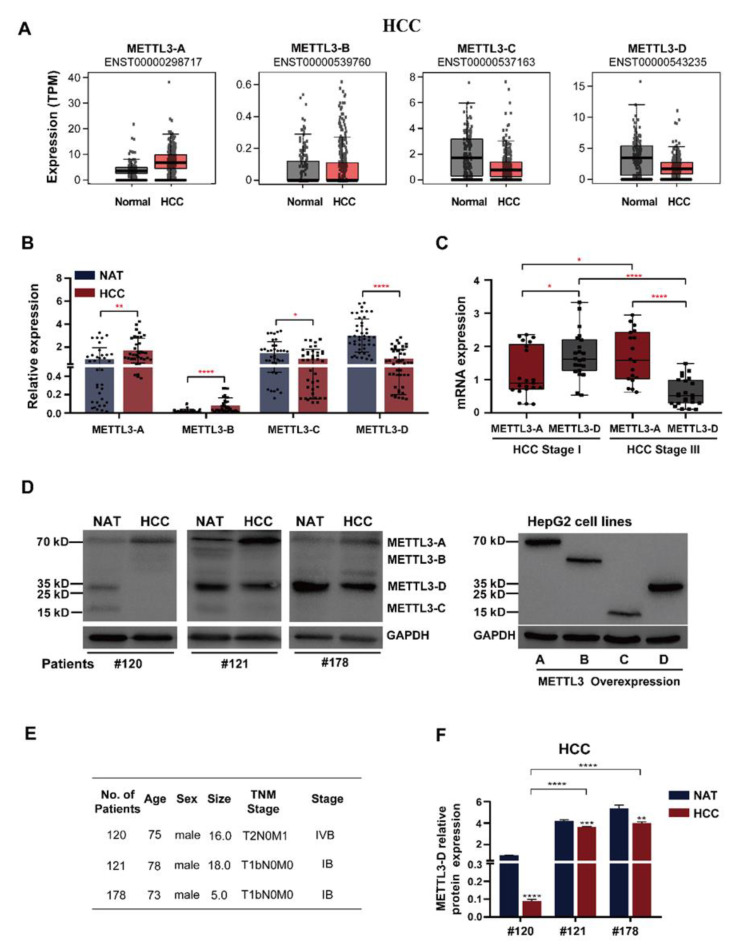
METTL3-D is downregulated in HCC and correlates with tumor stage. (**A**) mRNA levels of METTL3-A, -B, -C, and -D in normal and HCC tissues from the GEPIA2 database. (**B**) RT–qPCR analyses of the mRNA levels of METTL3-A, -B, -C, and -D in NATs and tumor tissues from our patients. The clinical characteristics of all the patients are listed in Table S1. (**C**) mRNA levels of METTL3-A and METTL3-D are correlated with tumor stage. The mRNA levels were analyzed by RT–qPCR, and patients were grouped as early-stage HCC (Stage I) and late-stage HCC (Stage III). (**D**) The protein levels of METTL3-A were upregulated in HCC tumor tissues, while METTL3-D was downregulated. Right, Western blot analysis of Flag-tagged METTL3 variants on the pcDNA3 vector in HepG2 cells using the anti-FLAG antibody. Left, Western blot analysis of the endogenous METTL3 variants in HCC patient NATs and tumor tissues using the anti-METTL3 antibody. (**E**) Information concerning patients in panel (**D**). (**F**) The protein levels of METTL3-A and METTL3-D in patients were correlated with their tumor stages. The data are expressed as means ± SD. * *p* < 0.05, ** *p* < 0.01, *** *p* < 0.001, **** *p* < 0.0001 (*t* test). Black and red asterisks have the same meaning.

To confirm the above pattern in a larger pool of patient samples, we first analyzed a set of TCGA data containing 369 HCC and 160 normal liver samples and observed that METTL3-A was upregulated in HCC samples, METTL3-B was not significantly changed, and METTL3-C and D were significantly downregulated (Figure 2A). Second, we tested our twenty paired HCC patient samples using RT-qPCR (Figure 2B). Consistent with the TCGA data, METTL3-A was significantly elevated in our HCC patient livers compared with their NATs, while METTL3-D was significantly decreased. By contrast, we observed a significant increase in METTL3-B and no obvious change in METTL3-C in our HCC patients. The reason may be the inaccuracy of amplification of lower level RNAs or unpaired samples from the TCGA database.

Next, we asked whether the levels of METTL3-A and METTL3-D were correlated with the TNM stages of HCC patients. The twenty patients we investigated were classified into two groups according to their developmental stages of tumors (Table S1). METTL3-D was at a relatively higher level in earlier tumor stage patients than in later stage patients. By contrast, METTL3-D was at a significantly lower level in later stage patients (Figure 2C).

To detect proteins translated from those splice variants, we first constructed overexpression (OE) plasmids of the four METTL3 isoforms using FLAG-tag and expressed them in HepG2 cells, which were analyzed by Western blotting and used as migration markers for in vivo METTL3 variants (Figure 2D right). Using another three paired HCC patient samples, we found that METTL3-A protein signals were obviously increased in tumor tissues compared with those in their NATs, while METTL3–D protein signals were significantly decreased in tumor tissues (Figure 2D left). The signals of METTL3-B and -C were weak and difficult to differentiate between samples. These findings are consistent with the above RNA analyses and strongly demonstrate that the splice variant METTL3-D is translated in both normal and tumor tissues and its protein level is significantly decreased in HCC tumors. Additionally, the protein levels of METTL3-D in these three patients were negatively correlated with the tumor stage (Figure 2E), in which patients #121 and #178 were at stage I and had much more METTL3-D proteins than patient #120 (Figure 2F).

Taken together, these results strongly demonstrate that METTL3-D is negatively correlated with HCC malignancy and is downregulated in HCC patient tumor tissues, suggesting that METTL3-D could be a tumor suppressor in HCC.

### 3.3. Expression of METTL3-D Decreases m^6^A Modification

**Figure 3:**
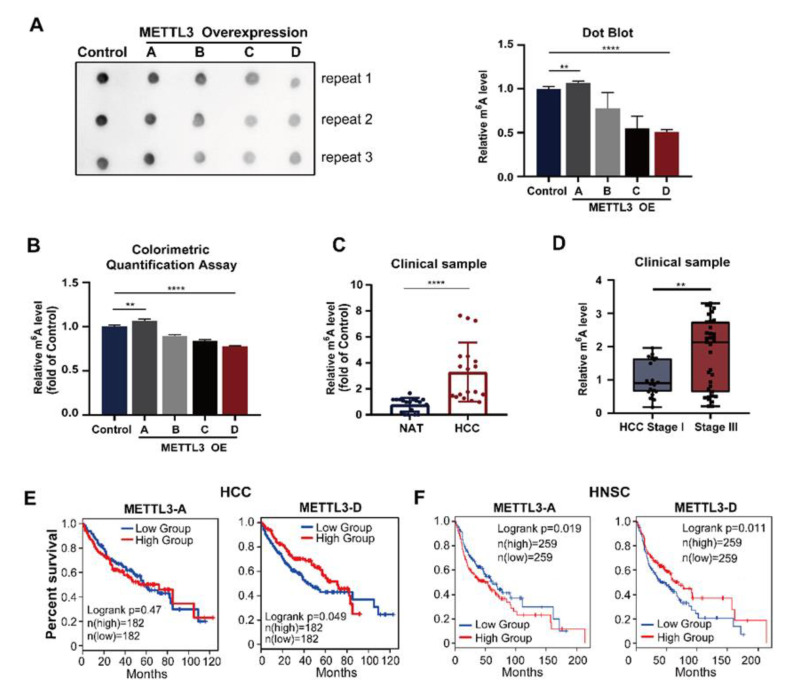
METTL3-D mediates RNA m^6^A modification in HCC cells. (A, B) OE of the METTL3 splice variants (-B, -C, and -D) decreases the m^6^A levels of total RNAs in HepG2 cells, while OE of METTL3-A increases the m^6^A levels. (**A**) Dot blot assay. (**B**) Colorimetric m^6^A quantification assay. The signal intensities of dot blots were quantitated by ImageJ. (**C**) Elevation of the m^6^A levels in HCC tumor tissues. m^6^A of mRNAs was evaluated using the colorimetric m^6^A quantification assay. (**D**) Significant correlation between the m^6^A levels and tumor stages among HCC patients. The m^6^A levels of total RNA from each patient were analyzed by the dot blot assay and quantitated by ImageJ. (**E**) HCC patients with higher levels of METTL3-D had longer overall survival times (TCGA data). (**F**) HNSC patients with higher levels of METTL3-D had longer overall survival times (TCGA data). The data are expressed as means ± SD. ** *p* < 0.01, **** *p* < 0.0001 (*t* test).

Because the splice variants METTL3-B, -C, and -D lack the adenosine methyltransferase catalytic domain MT-A70 (Figure 1A), we next evaluated their effects on RNA m^6^A modification. Dot blot analysis revealed that the m^6^A level of total RNAs in HepG2 cells was slightly increased when METTL3-A (WT) was overexpressed and significantly decreased when the three splice variants, particularly the METTL3-D variant, were overexpressed (Figure 3A,B).

Second, we detected m^6^A of mRNAs from 20 HCC patient samples and found that their levels in tumor samples were significantly higher than those in their corresponding NAT samples (Figure 3C). Further analysis revealed that the m^6^A levels were positively correlated with the tumor stages of patients; the later the stage was, the higher the level of m^6^A was (Figure 3D). Taken together, these results suggest that the presence of splice variants of METTL3 (particularly METTL3-D) significantly reduces the level of m^6^A modification in normal tissues, while downregulation of METTL3-D in HCC tumors results in increased levels of m^6^A modification.

### 3.4. METTL3-D Levels Are Inversely Correlated with Patient Survival in Multiple Cancers

The available TCGA data uncovered that the levels of METTL3-A were upregulated in most other solid tumors (Figure S5A), while the levels of the METTL3-D variant were downregulated (Figure S5B), showing a similar pattern to those in HCC and suggesting that aberrant splicing of METTL3 is a common feature in solid tumors.

**Figure 4:**
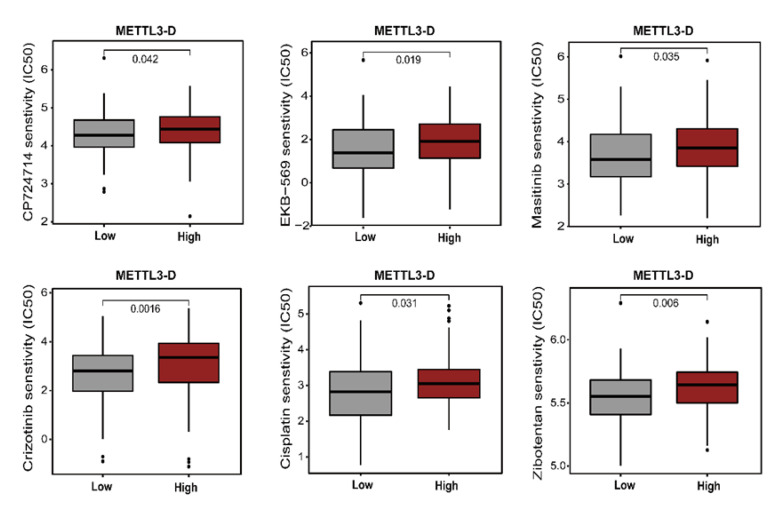
An illustration of the relationship between METTL3-D expression and expected medication response (TCGA data).

Further analyses of TCGA survival data revealed that HCC patients with lower levels of METTL3-D exhibited shorter overall survival than patients whose tumors had higher levels of METTL3-D (Figure 3E). TCGA data also indicated that HNSC patients with higher levels of METTL3-D in their tumors had significantly longer overall survival (Figure 3F). A similar pattern was also observed in patients with other solid tumors, such as LUAD, LUSC, BLCA, and PAAD, although statistically, their differences were not significant (Figure S6). Additionally, METTL3-D expression was positively correlated with drug response in patients treated with different tyrosine kinase inhibitors and other common anti-cancer drugs (Figure 4).

### 3.5. METTL3-D Inhibits the Proliferation, Migration, and Invasion of HCC Cells

**Figure 5:**
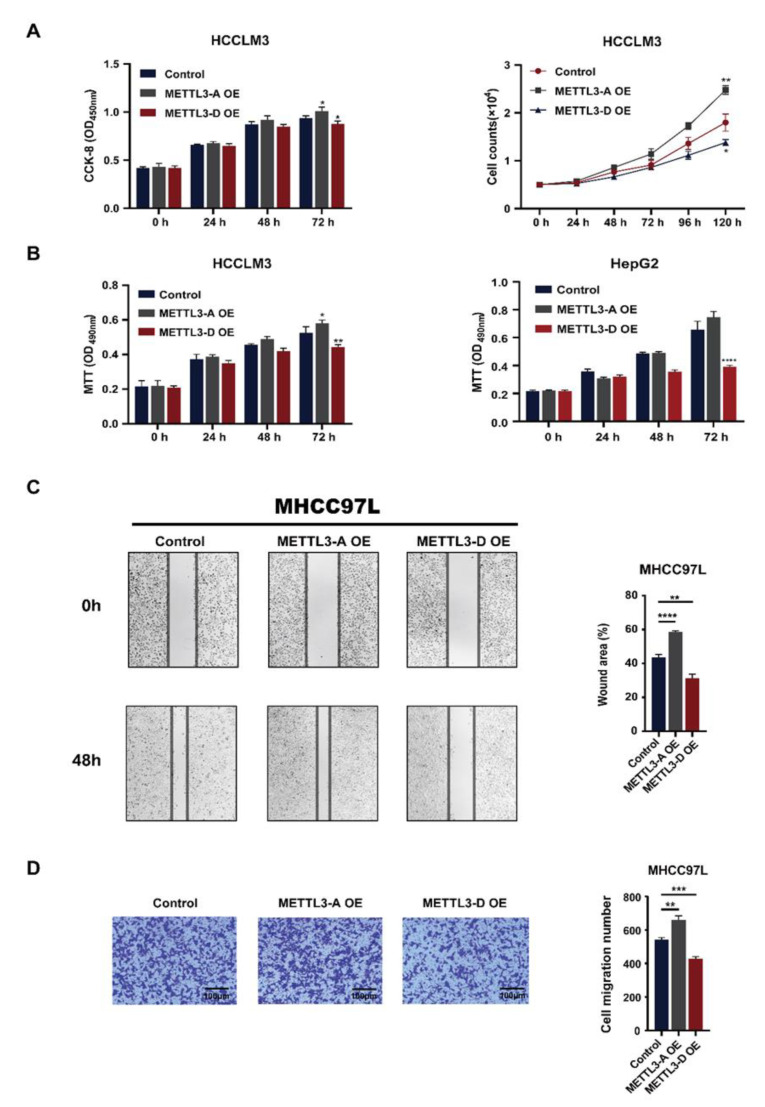
METTL3-D inhibits the proliferation, migration, and invasion of HCC cells. (**A**) Expression of METTL3-D decreases the proliferation rate of HCCLM3 cells. Left, CCK-8 assay analysis; right, cell counting assay analysis. (**B**) The MTT assay showed that METTL3-D expression decreases the proliferation rate of HCCLM3 (left) and HepG2 (right) cells. (**C**) Expression of METTL3-D inhibits the migration of MHCC97L cells. Representative images are shown at 0 h and 48 h after transfections. (**D**) Expression of METTL3-D inhibits the invasion ability of MHCC97L cells. The data are expressed as means ± SD. * *p* < 0.05, ** *p* < 0.01, *** *p* < 0.001, **** *p* < 0.0001 (*t* test).

Consistent with previous studies showing that METTL3 promotes the survival and invasion of HCC cells [31,46], we found that OE of canonical full-length METTL3-A facilitated the growth and proliferation of HCCLM3 cells, as demonstrated by various assays, including regular cell counting, the CCK-8 assay (Figure 5A), and the MTT assay (Figure 5B, left). By contrast, OE of the METTL3-D variant suppressed cell growth and proliferation (Figure 5A,B left). A similar pattern was also observed in HepG2 cells (Figure 5B right).

Furthermore, the wound healing assay revealed that the migration ability of HCC cells was enhanced by METTL3-A but inhibited by METTL3-D in both MHCC97L (Figure 5C) and HepG2 (Figure S7A) cells. Similarly, the invasion abilities of these two HCC cells, as demonstrated by the Transwell assay, were enhanced by METTL3-A but inhibited by METTL3-D (Figure 5D and Figure S7B). These results demonstrate that, unlike canonical full-length METTL3, the METTL3-D variant is a tumor suppressor that inhibits HCC proliferation, migration, and invasion.

### 3.6. METTL3-D Has an Opposite Function to Downstream Genes

**Figure 6:**
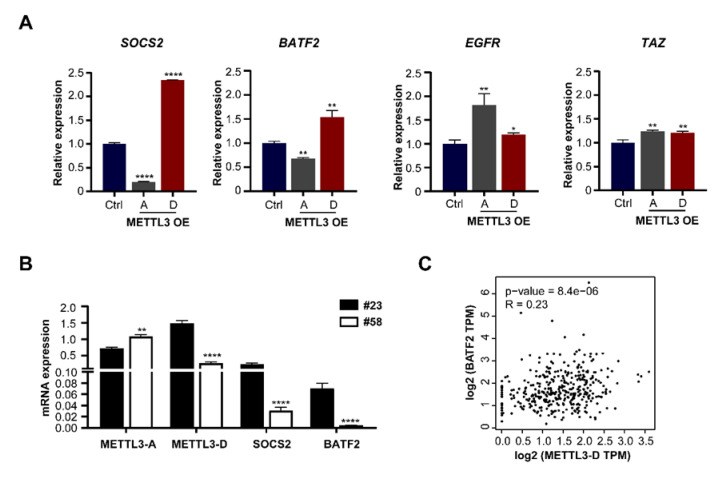
The METTL3-D splice variant has an opposite function to that of the downstream genes of the canonical full-length METTL3-A. (**A**) RT–qPCR analyses of the mRNA levels of downstream target genes in METTL3-A- or METTL3-D-overexpressing HepG2 cells. SOCS2 and BATF2 were previously reported as mRNA stability-decreased target genes of METTL3, while EGFR and TAZ were previously reported as mRNA translation-affected target genes [31,32,33]. (**B**) mRNA levels of SOCS2 and BATF2 in two HCC patients. Compared with patient #58, patient #23 had higher levels of METTL3-D and higher levels of SOCS2 and BATF2. (**C**) Correlation coefficient between METTL3-D and BATF2 expression in HCC patients. The original data were downloaded from GEPIA2. The data in the bar graphs are expressed as means ± SD. * *p* < 0.05, ** *p* < 0.01, **** *p* < 0.0001 (*t* test).

Previous studies have shown that the expression of many downstream genes is changed following the upregulation of METTL3-A in multiple cancers [31,32,33,47,48] by mechanistic regulation of their mRNA stabilities or translation due to increased m^6^A modification [49,50]. For example, METTL3-mediated m^6^A modification destabilizes SOCS2 mRNA in HCC and BATF2 mRNA in gastric cancer [32] and promotes the translation of epidermal growth factor receptor (EGFR) and the Hippo pathway effector TAZ in human lung cancer cells [33]. To investigate whether the METTL3-D variant also has effects, we measured the mRNA levels of those genes in METTL3-A- or METTL3-D-overexpressing HepG2 cells by RT–qPCR. Consistent with previous studies, the mRNA levels of BATF2 and SOCS2 were decreased following OE of METTL3-A in HepG2 cells. By contrast, the mRNA levels of BATF2 and SOCS2 were significantly increased following OE of the METTL3-D variant (Figure 6A, left). Additionally, the mRNA levels of EGFR and TAZ were not decreased following OE of METTL3-A and were slightly increased following OE of METTL3-D, findings that are consistent with previous findings that increased m^6^A modification affects their translation abilities but not their RNA stabilities (Figure 6A, right).

We also found that the mRNA levels of SOCS2 and BATF2 were significantly higher in patient #23 than in patient #58, in which the level of METTL3-D showed the same pattern (Figure 6B). Further analysis of TCGA data confirmed that the mRNA level of METTL3-D was positively correlated with that of BATF2 in HCC samples (Figure 6C). Taken together, these results demonstrate that the METTL3-D variant has an opposite function to downstream genes of METTL3-A in HCC cells and patients.

## 4. Discussion

Alternative splicing produces multiple mRNA isoforms from one gene and enhances transcriptome plasticity and proteome diversity, which are crucial functions to regulate eukaryotic gene expression, particularly in mammals. Alternative splicing-generated splice variants of oncogenes have been associated with the development of many cancers [10,14,16,18]. METTL3 is the kernel of the m^6^A methyltransferase complex and affects the occurrence and development of multiple cancers [31,33,47]. METTL3 is upregulated in HCC patient tumor tissues, leading to increased mRNA m^6^A modification and enhanced progression of HCC [31]. Based on at least three lines of evidence, we identified a splice variant of METTL3, METTL3-D, which has an opposite function to canonical full-length METTL3 (named METTL3-A here). (1) Expression analyses of mRNAs and proteins revealed that the METTL3-D level is opposite that of METTL3-A. The level of METTL3-D is higher than that of METTL3-A in human normal liver tissues and HCC patient NATs but is lower than that of METTL3-A in HCC cell lines and patient tumors. (2) In contrast to the promotion of m^6^A modification and mRNA decay/stability of downstream target genes by METTL3-A, METTL3-D inhibits cellular m^6^A modification and protects the mRNA decay/stability of downstream genes. (3) METTL3-D is negatively associated with the malignancy of HCC tumors, including its positive correlation with longer patient survival times and negative correlations with the growth, migration, and invasion of HCC cells. Therefore, we propose a novel inactivation mechanism of HCC oncogenes using two steps of posttranscriptional regulation, alternative splicing and RNA modification, in which the alternatively spliced variant of METTL3 antagonizes the function of its canonical full-length isoform, decreasing the m^6^A modification and thereafter altering the mRNA decay/stability of HCC oncogenes.

Compared with canonical full-length splicing isoforms, alternatively spliced variants often introduce a PTC in their mRNAs, such as METTL3-B, -C, and -D. Most PTC-containing mRNAs are rapidly degraded via an mRNA surveillance mechanism, nonsense-mediated mRNA decay (NMD) [48]. However, several PTC-containing mRNAs generated by aberrant splicing evade the NMD pathway and are translated into truncated proteins in various diseases, such as β-globin in β-thalassemia and BRCA1 in breast cancer [49,50]. Here, the mRNA level of METTL3-D was higher than that of METTL3-A in the normal human liver. Additionally, the truncated METTL3-D-encoding protein was stable in HCC patient NATs and tumors (Figure 2D). Global mRNA analysis also showed that the METTL3-D level is similar or even higher than that of METTL3-A in most human tissues (Figure 1B). These results indicate that the truncated METTL3-D protein, lacking the (N6-adenosine)-methyltransferase domain, is a critical regulator of tissue homeostasis by competing with the full-length METTL3-A protein. Aberrant splicing decreasing the level of METTL3-D substantially contributes to the tumor progression of HCC.

Understanding the cellular and molecular mechanisms driving HCC has made considerable progress in the past several years [51]. However, many studies have ignored the importance of alternative splicing as a source of novel HCC prognostic markers and disease targets, although aberrant expression could act as oncogenic drivers and passenger factors in other cancers [52]. Mutations in the *cis*-elements of pre-mRNAs, core spliceosomal proteins, and splicing regulators have been extensively reported in HCC [53,54,55]. Presently, which mutations cause the aberrant splicing of METTL3 in HCC are unknown, and their regulatory mechanism will be of interest in future studies.

HCC patients with higher METTL3-D and lower METTL3-A have longer overall survival times. Therefore, METTL3-D and METTL3-A could be used as predictive factors for postoperative HCC survival. Recently, antisense oligonucleotides (ASOs) blocking RNA *cis*-elements and/or altering RNA structures have been extensively studied for their modulation of splicing in vivo [56,57]. In the future, isoform switching from METTL3-A to METTL3-D using ASOs or other methods could be a potential method for HCC therapy. Additionally, this study reveals the function of a METTL3 splice variant, suggesting that comprehensive understanding of other METTL3 splice variants would be helpful to eventually understand HCC development and therapy.

## Data Availability

HepG2 RNA sequencing data have been deposited to NCBI under accession number GSE189372.

## Supplementary Materials

The following supporting information can be downloaded at: https://www.mdpi.com/article/10.3390/genes13040669/s1, Figure S1: Alternatively Spliced Isoforms of METTL3 in Human Tissues.; Figure S2: Primary Sequences of the Proteins Encoded by the Four METTL3 Isoforms; Figure S3: Validation of the METTL3 Splice Variants; Figure S4: The Splice Variant Mettl3-b is Conserved in Mice and Humans; Figure S5: mRNA Levels of METTL3-A and METTL3-D in Different Cancers; Figure S6: Correlation between the Levels of the METTL3-D Variant and Patient Survival in Multiple Cancers; Figure S7: METTL3-D Inhibits the Migration and Invasion of HepG2 Cells. Table S1: Clinical Characteristics of Patients; Table S2: List of Primers Used in This Study.

## References

[1] Sharp P.A. (2009). The centrality of RNA. Cell.

[2] Gupta S., Zink D., Korn B., Vingron M., Haas S.A. (2004). Genome wide identification and classification of alternative splicing based on EST data. Bioinformatics.

[3] Wahl M.C., Will C.L., Lührmann R. (2009). The spliceosome: Design principles of a dynamic RNP machine. Cell.

[4] Wang E.T., Sandberg R., Luo S., Khrebtukova I., Zhang L., Mayr C., Kingsmore S.F., Schroth G.P., Burge C.B. (2008). Alternative isoform regulation in human tissue transcriptomes. Nature.

[5] Stamm S., Ben-Ari S., Rafalska I., Tang Y., Zhang Z., Toiber D., Thanaraj T., Soreq H. (2005). Function of alternative splicing. Gene.

[6] David C.J., Manley J.L. (2010). Alternative pre-mRNA splicing regulation in cancer: Pathways and programs unhinged. Genes Dev..

[7] Wang Y., Bao Y., Zhang S., Wang Z. (2020). Splicing dysregulation in cancer: From mechanistic understanding to a new class of therapeutic targets. Sci. China Life Sci..

[8] Huun J., Gansmo L.B., Mannsåker B., Iversen G.T., Øvrebø J.I., Lønning P.E., Knappskog S. (2017). Impact of the MDM2 splice-variants MDM2-A, MDM2-B and MDM2-C on cytotoxic stress response in breast cancer cells. BMC Cell Biol..

[9] Mercatante D.R., Mohler J.L., Kole R. (2002). Cellular response to an antisense-mediated shift of Bcl-x pre-mRNA splicing and antineoplastic agents. J. Biol. Chem..

[10] Lin K.-T., Ma W.K., Scharner J., Liu Y.-R., Krainer A.R. (2018). A human-specific switch of alternatively spliced AFMID isoforms contributes to TP53 mutations and tumor recurrence in hepatocellular carcinoma. Genome Res..

[11] Qi Y., Yu J., Han W., Fan X., Qian H., Wei H., Yi-hsuan S.T., Zhao J., Zhang W., Liu Q. (2016). A splicing isoform of TEAD4 attenuates the Hippo–YAP signalling to inhibit tumour proliferation. Nat. Commun..

[12] Li S., Hu Z., Zhao Y., Huang S., He X. (2019). Transcriptome-wide analysis reveals the landscape of aberrant alternative splicing events in liver cancer. Hepatology.

[13] Zhu G.-Q., Zhou Y.-J., Qiu L.-X., Wang B., Yang Y., Liao W.-T., Luo Y.-H., Shi Y.-H., Zhou J., Fan J. (2019). Prognostic alternative mRNA splicing signature in hepatocellular carcinoma: A study based on large-scale sequencing data. Carcinogenesis.

[14] Zhang L., Liu X., Zhang X., Chen R. (2016). Identification of important long non-coding RNAs and highly recurrent aberrant alternative splicing events in hepatocellular carcinoma through integrative analysis of multiple RNA-Seq datasets. Mol. Genet. Genom..

[15] Jin H., Wang C., Jin G., Ruan H., Gu D., Wei L., Wang H., Wang N., Arunachalam E., Zhang Y. (2017). Regulator of calcineurin 1 gene isoform 4, down-regulated in hepatocellular carcinoma, prevents proliferation, migration, and invasive activity of cancer cells and metastasis of orthotopic tumors by inhibiting nuclear translocation of NFAT1. Gastroenterology.

[16] Snider N.T., Altshuler P.J., Wan S., Welling T.H., Cavalcoli J., Omary M.B. (2014). Alternative splicing of human NT5E in cirrhosis and hepatocellular carcinoma produces a negative regulator of ecto-5′-nucleotidase (CD73). Mol. Biol. Cell.

[17] Wang Z., Li S.S.-C. (2010). Numb: A new player in EMT. Cell Adhes. Migr..

[18] Lu Y., Xu W., Ji J., Feng D., Sourbier C., Yang Y., Qu J., Zeng Z., Wang C., Chang X. (2015). Alternative splicing of the cell fate determinant Numb in hepatocellular carcinoma. Hepatology.

[19] Meyer K.D., Saletore Y., Zumbo P., Elemento O., Mason C.E., Jaffrey S.R. (2012). Comprehensive analysis of mRNA methylation reveals enrichment in 3′ UTRs and near stop codons. Cell.

[20] Yang Y., Hsu P.J., Chen Y.-S., Yang Y.-G. (2018). Dynamic transcriptomic m^6^A decoration: Writers, erasers, readers and functions in RNA metabolism. Cell Res..

[21] Dominissini D., Moshitch-Moshkovitz S., Schwartz S., Salmon-Divon M., Ungar L., Osenberg S., Cesarkas K., Jacob-Hirsch J., Amariglio N., Kupiec M. (2012). Topology of the human and mouse m^6^A RNA methylomes revealed by m 6 A-seq. Nature.

[22] He P.C., He C. (2021). m^6^A RNA methylation: From mechanisms to therapeutic potential. EMBO J..

[23] Hsu P.J., Zhu Y., Ma H., Guo Y., Shi X., Liu Y., Qi M., Lu Z., Shi H., Wang J. (2017). Ythdc2 is an N 6-methyladenosine binding protein that regulates mammalian spermatogenesis. Cell Res..

[24] Li L., Zang L., Zhang F., Chen J., Shen H., Shu L., Liang F., Feng C., Chen D., Tao H. (2017). Fat mass and obesity-associated (FTO) protein regulates adult neurogenesis. Hum. Mol. Genet..

[25] Han X., Wang M., Zhao Y.-L., Yang Y., Yang Y.-G. (2020). RNA methylations in human cancers. Seminars in Cancer Biology.

[26] Bokar J.A., Rath-Shambaugh M.E., Ludwiczak R., Narayan P., Rottman F. (1994). Characterization and partial purification of mRNA N6-adenosine methyltransferase from HeLa cell nuclei. Internal mRNA methylation requires a multisubunit complex. J. Biol. Chem..

[27] Bujnicki J.M., Feder M., Radlinska M., Blumenthal R.M. (2002). Structure prediction and phylogenetic analysis of a functionally diverse family of proteins homologous to the MT-A70 subunit of the human mRNA: m^6^A methyltransferase. J. Mol. Evol..

[28] Bokar J., Shambaugh M., Polayes D., Matera A., Rottman F. (1997). Purification and cDNA cloning of the AdoMet-binding subunit of the human mRNA (N6-adenosine)-methyltransferase. RNA.

[29] Batista P.J., Molinie B., Wang J., Qu K., Zhang J., Li L., Bouley D.M., Lujan E., Haddad B., Daneshvar K. (2014). m^6^A RNA modification controls cell fate transition in mammalian embryonic stem cells. Cell Stem Cell.

[30] Wang Y., Li Y., Toth J.I., Petroski M.D., Zhang Z., Zhao J.C. (2014). N 6-methyladenosine modification destabilizes developmental regulators in embryonic stem cells. Nat. Cell Biol..

[31] Chen M., Wei L., Law C.T., Tsang F.H.C., Shen J., Cheng C.L.H., Tsang L.H., Ho D.W.H., Chiu D.K.C., Lee J.M.F. (2018). RNA N6-methyladenosine methyltransferase-like 3 promotes liver cancer progression through YTHDF2-dependent posttranscriptional silencing of SOCS2. Hepatology.

[32] Xie J.-W., Huang X.-B., Chen Q.-Y., Ma Y.-B., Zhao Y.-J., Liu L.-C., Wang J.-B., Lin J.-X., Lu J., Cao L.-L. (2020). m^6^A modification-mediated BATF2 acts as a tumor suppressor in gastric cancer through inhibition of ERK signaling. Mol. Cancer.

[33] Lin S., Choe J., Du P., Triboulet R., Gregory R.I. (2016). The m^6^A methyltransferase METTL3 promotes translation in human cancer cells. Mol. Cell.

[34] Ping X.-L., Sun B.-F., Wang L., Xiao W., Yang X., Wang W.-J., Adhikari S., Shi Y., Lv Y., Chen Y.-S. (2014). Mammalian WTAP is a regulatory subunit of the RNA N6-methyladenosine methyltransferase. Cell Res..

[35] Liu N., Dai Q., Zheng G., He C., Parisien M., Pan T. (2015). N 6-methyladenosine-dependent RNA structural switches regulate RNA–protein interactions. Nature.

[36] Sung H., Ferlay J., Siegel R.L., Laversanne M., Soerjomataram I., Jemal A., Bray F. (2021). Global cancer statistics 2020: GLOBOCAN estimates of incidence and mortality worldwide for 36 cancers in 185 countries. CA A Cancer J. Clin..

[37] Chen M., Wong C.-M. (2020). The emerging roles of N6-methyladenosine (m^6^A) deregulation in liver carcinogenesis. Mol. Cancer.

[38] Zhou Y., Yin Z., Hou B., Yu M., Chen R., Jin H., Jian Z. (2019). Expression profiles and prognostic significance of RNA N6-methyladenosine-related genes in patients with hepatocellular carcinoma: Evidence from independent datasets. Cancer Manag. Res..

[39] Dobin A., Davis C.A., Schlesinger F., Drenkow J., Zaleski C., Jha S., Batut P., Chaisson M., Gingeras T.R. (2013). STAR: Ultrafast universal RNA-seq aligner. Bioinformatics.

[40] Livak K.J., Schmittgen T.D. (2001). Analysis of relative gene expression data using real-time quantitative PCR and the 2(-Delta Delta C(T)) method. Methods.

[41] Cunningham F., Achuthan P., Akanni W., Allen J., Amode M.R., Armean I.M., Bennett R., Bhai J., Billis K., Boddu S. (2019). Ensembl 2019. Nucleic Acids Res..

[42] Buels R., Yao E., Diesh C.M., Hayes R.D., Munoz-Torres M., Helt G., Goodstein D.M., Elsik C.G., Lewis S.E., Stein L. (2016). JBrowse: A dynamic web platform for genome visualization and analysis. Genome Biol..

[43] Consortium E.P. (2012). An integrated encyclopedia of DNA elements in the human genome. Nature.

[44] Tang Z., Kang B., Li C., Chen T., Zhang Z. (2019). GEPIA2: An enhanced web server for large-scale expression profiling and interactive analysis. Nucleic Acids Res..

[45] Rasband W. (2018). WS 1997-2018. ImageJ.

[46] Pan F., Lin X., Hao L., Chu X., Wan H., Wang R. (2021). The role of RNA methyltransferase METTL3 in hepatocellular carcinoma: Results and perspectives. Front. Cell Dev. Biol..

[47] Liu L., Wu Y., Li Q., Liang J., He Q., Zhao L., Chen J., Cheng M., Huang Z., Ren H. (2020). METTL3 Promotes Tumorigenesis and Metastasis through BMI1 m^6^A Methylation in Oral Squamous Cell Carcinoma. Mol. Ther..

[48] Kurosaki T., Popp M.W., Maquat L.E. (2019). Quality and quantity control of gene expression by nonsense-mediated mRNA decay. Nat. Rev. Mol. Cell Biol..

[49] Supek F., Lehner B., Lindeboom R.G. (2020). To NMD or not to NMD: Nonsense-mediated mRNA decay in cancer and other genetic diseases. Trends Genet..

[50] Pawlicka K., Kalathiya U., Alfaro J. (2020). Nonsense-mediated mRNA decay: Pathologies and the potential for novel therapeutics. Cancers.

[51] Sia D., Villanueva A., Friedman S.L., Llovet J.M. (2017). Liver cancer cell of origin, molecular class, and effects on patient prognosis. Gastroenterology.

[52] Zhang Y., Qian J., Gu C., Yang Y. (2021). Alternative splicing and cancer: A systematic review. Signal Transduct. Target. Ther..

[53] Sen S. (2018). Aberrant pre-mRNA splicing regulation in the development of hepatocellular carcinoma. Hepatoma Res..

[54] Liu L., Xie S., Zhang C., Zhu F. (2014). Aberrant regulation of alternative pre-mRNA splicing in hepatocellular carcinoma. Crit. Rev. Eukaryot. Gene Expr..

[55] Lee S.E., Alcedo K.P., Kim H.J., Snider N.T. (2020). Alternative splicing in hepatocellular carcinoma. Cell. Mol. Gastroenterol. Hepatol..

[56] Singh N.N., Luo D., Singh R.N. (2018). Pre-mRNA splicing modulation by antisense oligonucleotides. Exon Skipping and Inclusion Therapies.

[57] Lim K.H., Han Z., Jeon H.Y., Kach J., Jing E., Weyn-Vanhentenryck S., Downs M., Corrionero A., Oh R., Scharner J. (2020). Antisense oligonucleotide modulation of non-productive alternative splicing upregulates gene expression. Nat. Commun..

