# Supplementary material for: An Alternatively Spliced Variant of METTL3 Mediates Tumor Suppression in Hepatocellular Carcinoma
Source: Genes (Basel). 2022 Apr 11;13(4):669. doi: 10.3390/genes13040669 (PMC9031889; doi:10.3390/genes13040669)
Supplement: Supplementary file 1 [file genes-13-00669-s001.zip › Table S2.pdf]

Table S2 List of primers used in this study.

Primers for qRT-PCR		
**Gene**	**Human/mouse**	**Sequence (5'-3')**
METTL3-A	Human	F: CTAAACCTAAGAATTTATAGAAG
		R: CCAACTACAATACAAATGTTTAT
METTL3-B	Human	F: CACTGCTTGGTTCGTTCCACCAG
		R: GAATCCCAACTACAATACAAATG
METTL3-C	Human	F: TGATTGAGCATCGGAACCAGCAA
		R: GCTCTAGGATCTCCTGACTGACC
METTL3-D	Human	F: CAACAGAGCAAGAAGGTCGGGCC
		R: CCATGGCCCTGCCTGTGACCCAG
MLF2	Human	F: GGAATCGGCTCTGGAAGGTG
		R: GCTCAGCTCTGGGATGATGT
NKD2	Human	F: ACAGGAGGTTGTCTGCACACG
		R: GACTTGAGGAACTGCTTCTCC
SOCS2	Human	F: TGCAAGGATAAGCGGACAGG
		R: CAGAGATGGTGCTGACGTGT
JUNB	Human	F: AGGCTCGGTTTCAGGAGTTT
		R: GAACAGCCCTTCTACCACGA
BMI1	Human	F: CATCCACAGTTTCCTCACATTTC
		R: GAAGTTGCTGATGACCCATTTAC
EGFR	Human	F: TCTGAGTGCAACCAGCAACA
		R: GTGGGGTCTGAGCTGTATCG
BATF2	Human	F: GCAGGGGTCTTCCTCTAAGC
		R: GCTGCTGAGAGAGCAGGTTT
GAPDH	Human	F: TCAAGTGGGGCGATGCTGGC
		R: TGGGGGCATCAGCAGAGGGG
Mettl3-a	Mouse	F: ACCGGAAGTGGCTTTTCATCTTG
		R: CCTTAAATCCAAGTGCCCAGAGT
Mettl3-b	Mouse	F: TCCACCTCAGGGCCATGGAATTG
		R: GTGGAACGAACCAAGCAGTGTTC
Gapdh	Mouse	F: TTCACCACCATGGAGAAGGC
		R: GGCATGGACTGTGGTCATGA
**Primers for PCR**		
METTL3-A	Human	F: GGAGAGAATGTCTAAACCTCTGG
		R: CCAACTACAATACAAATGTTTAT
METTL3-C	Human	F: AGTTGATTGAGCATCGGAACCAG
		R: GTTACACAAGATTGCAATTCTAG
METTL3-D	Human	F: GAACAACAGAGCAAGAAGGTCGG
		R: ATTGTACAAATATCACTCTTCAG
