# Supplementary figures and images for: An Alternatively Spliced Variant of METTL3 Mediates Tumor Suppression in Hepatocellular Carcinoma
Source: Genes (Basel). 2022 Apr 11;13(4):669. doi: 10.3390/genes13040669 (PMC9031889; doi:10.3390/genes13040669)

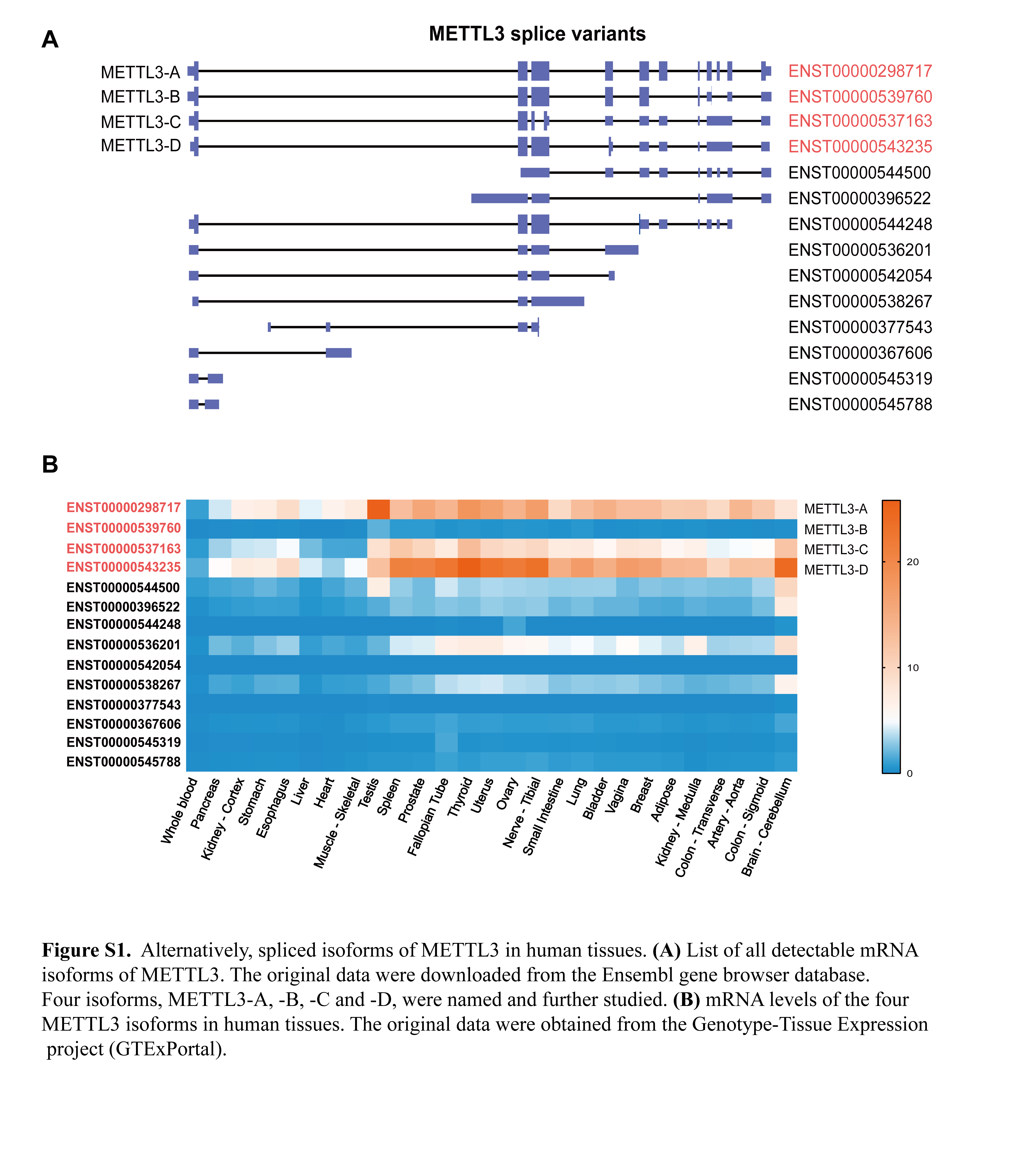

Supplement: Supplementary file 1 [file genes-13-00669-s001.zip › Figure S1.tif]

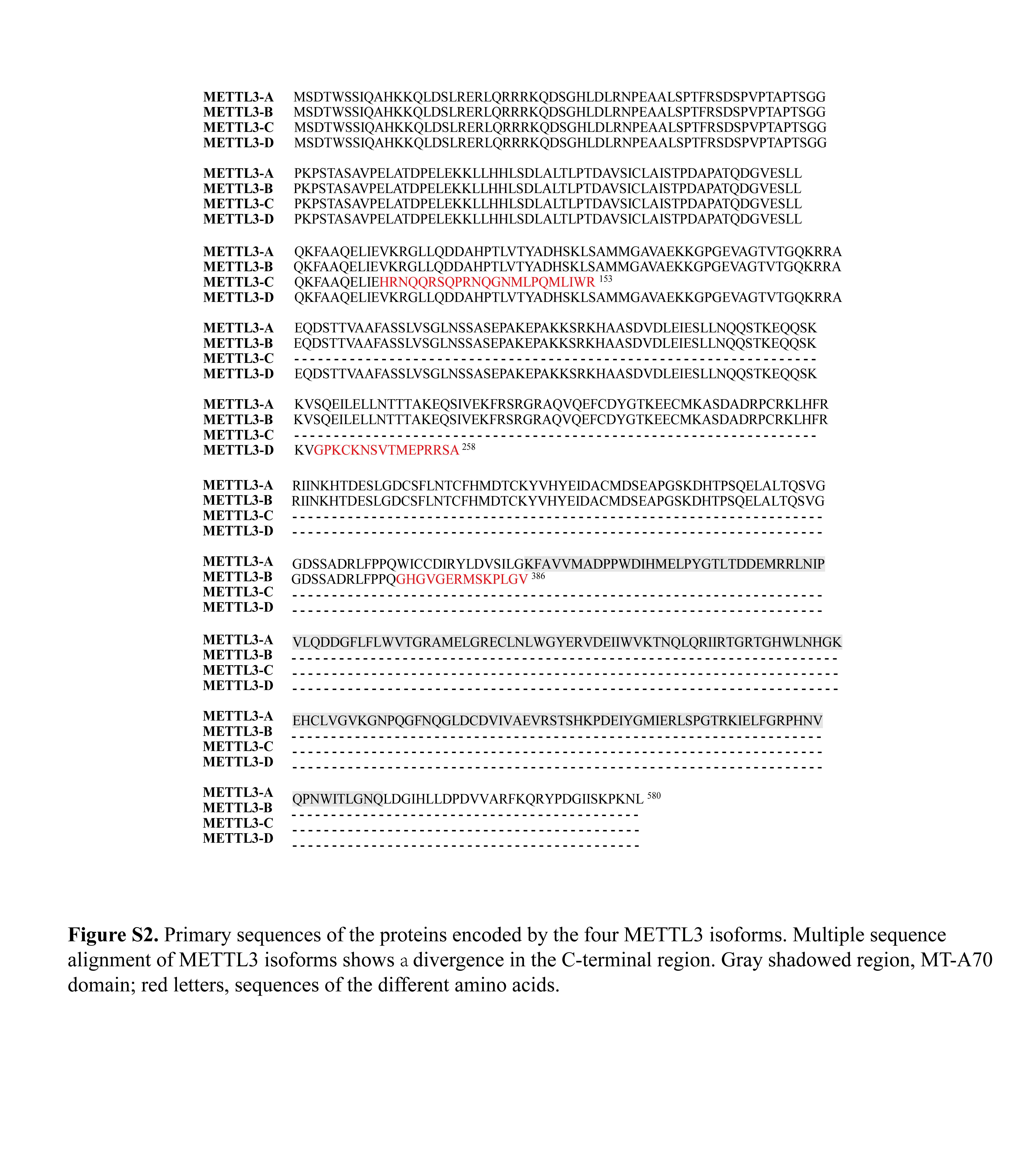

Supplement: Supplementary file 1 [file genes-13-00669-s001.zip › Figure S2.tif]

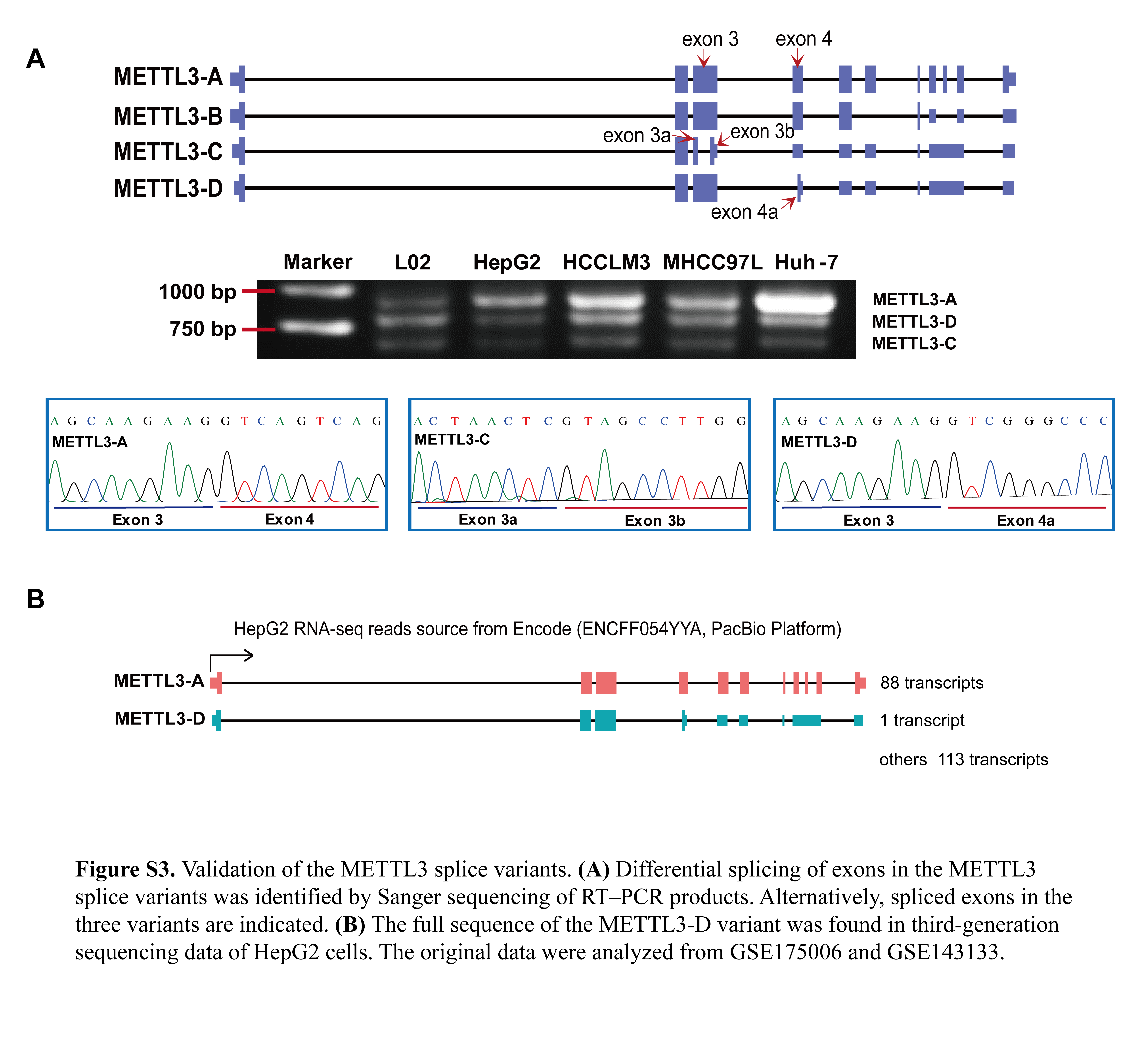

Supplement: Supplementary file 1 [file genes-13-00669-s001.zip › Figure S3.tif]

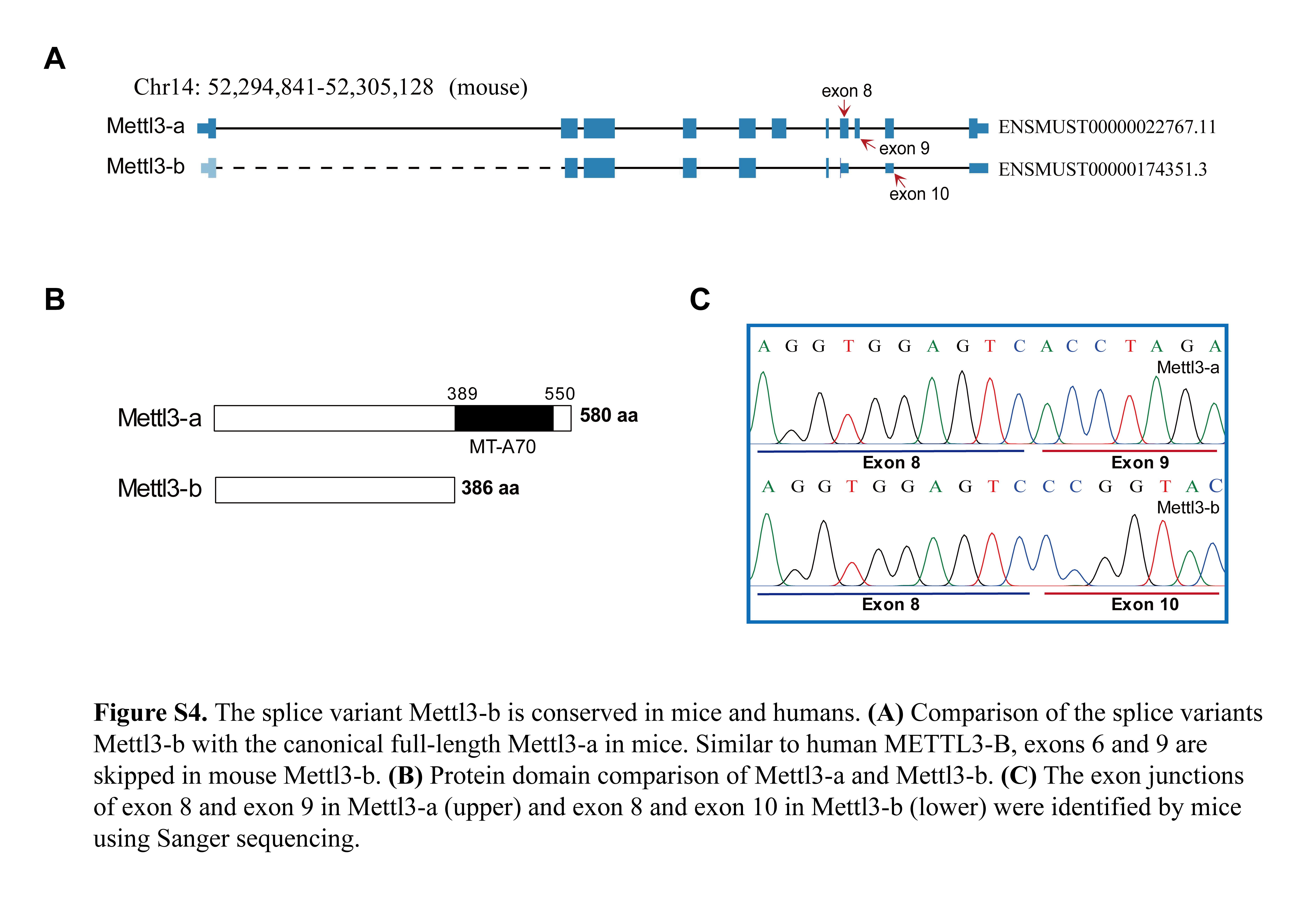

Supplement: Supplementary file 1 [file genes-13-00669-s001.zip › Figure S4.tif]

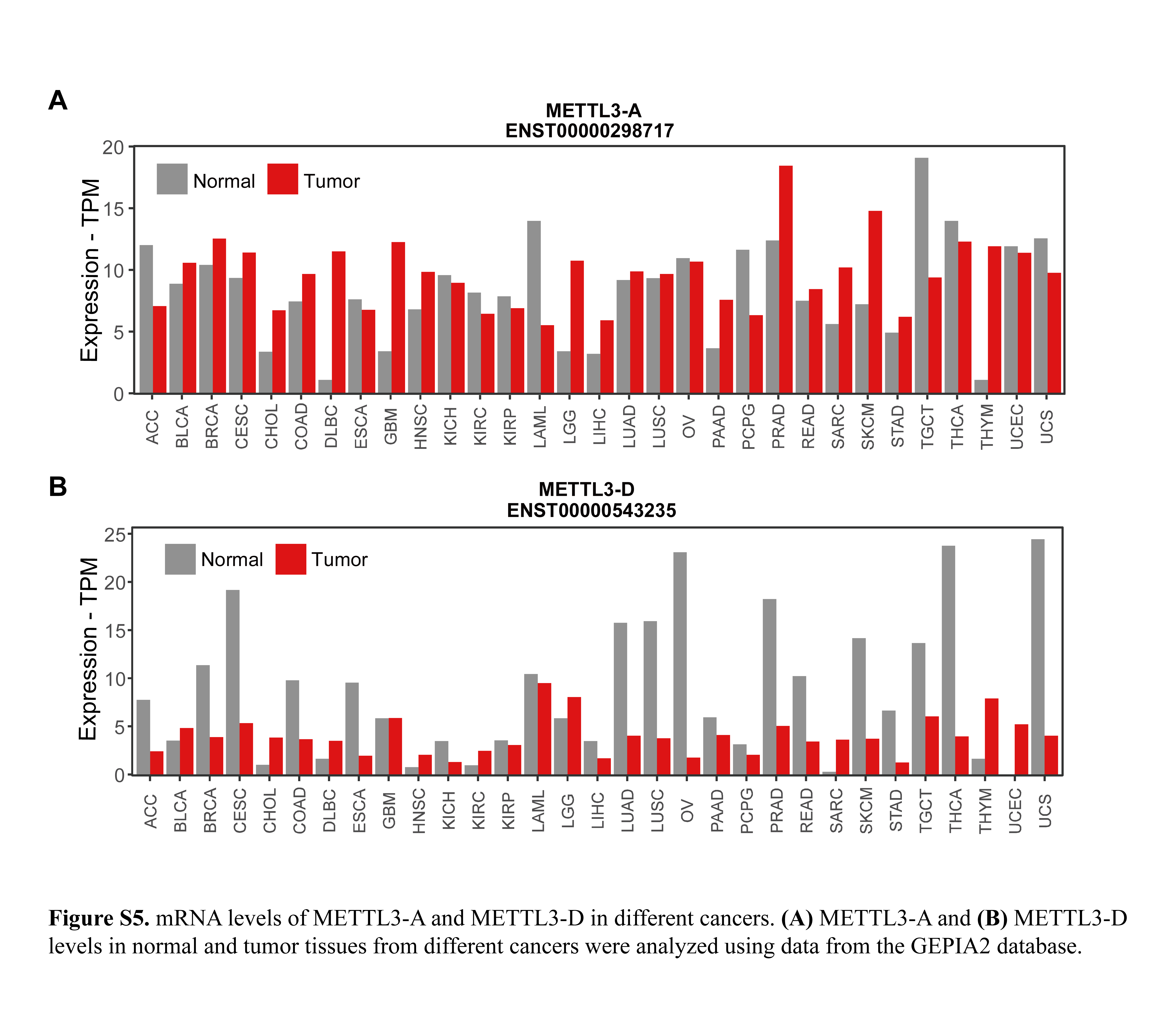

Supplement: Supplementary file 1 [file genes-13-00669-s001.zip › Figure S5.tif]

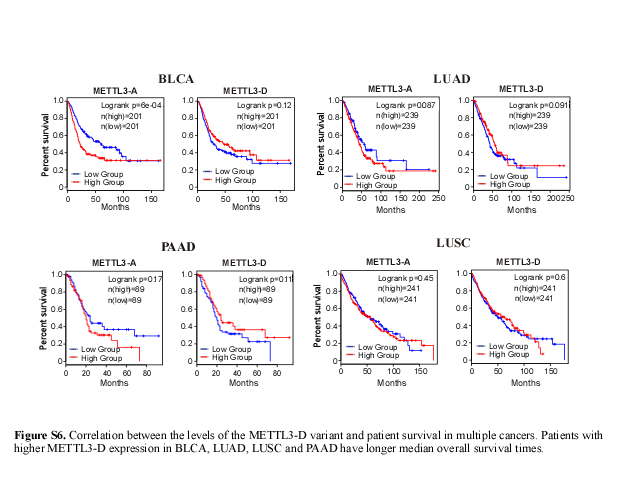

Supplement: Supplementary file 1 [file genes-13-00669-s001.zip › Figure S6.tif]

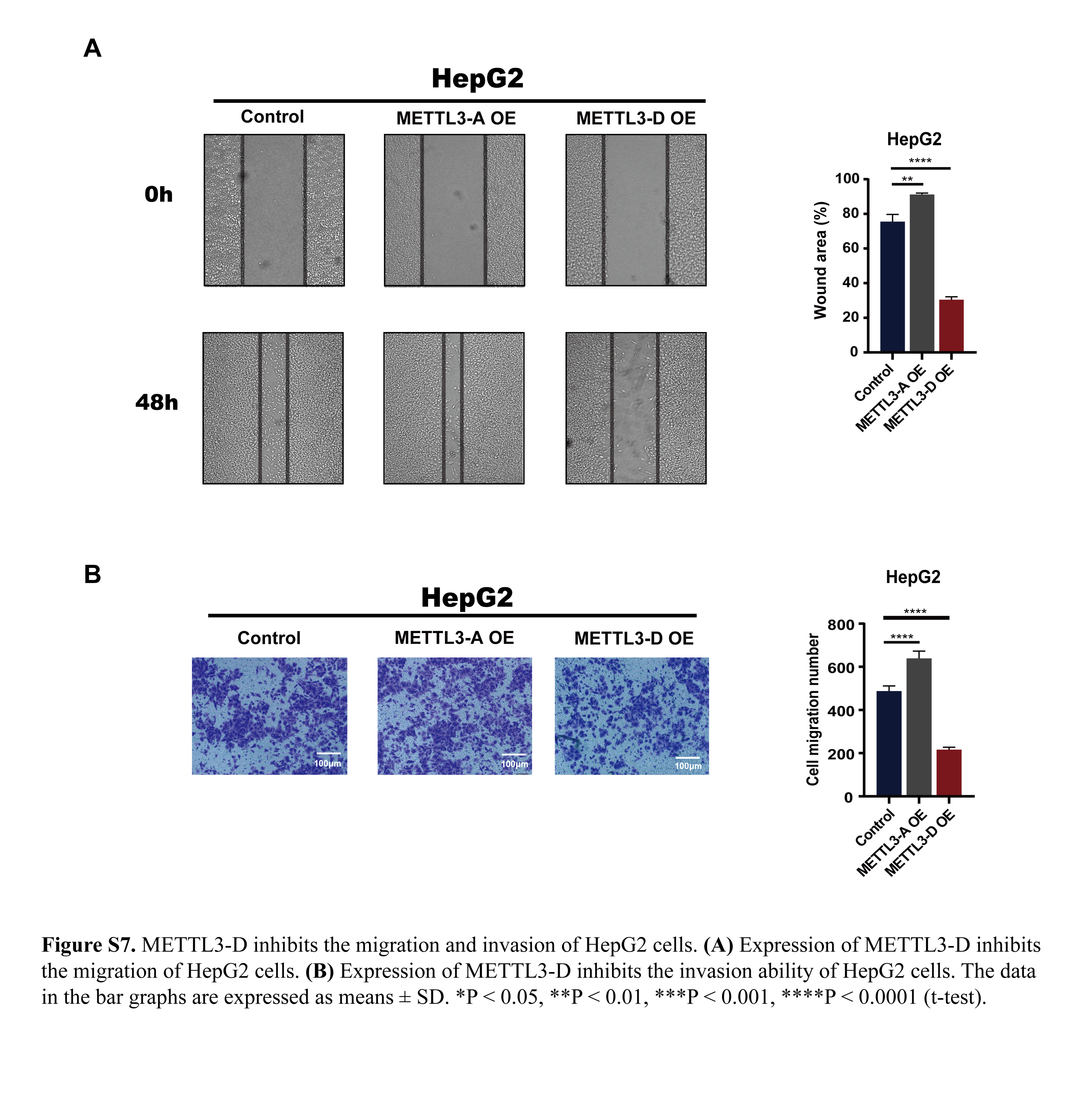

Supplement: Supplementary file 1 [file genes-13-00669-s001.zip › Figure S7.tif]
